# Ubiquitin-Proteasome System–Regulated Protein Degradation in Spermatogenesis

**DOI:** 10.3390/cells11061058

**Published:** 2022-03-21

**Authors:** Yi Xiong, Chao Yu, Qianting Zhang

**Affiliations:** 1 Zhejiang University-University of Edinburgh Institute (ZJU-UoE Institute), Zhejiang University School of Medicine, International Campus, Zhejiang University, 718 East Haizhou Rd, Haining 314400, China; yix.18@intl.zju.edu.cn; 2 Key Laboratory of Reproductive Dysfunction Management of Zhejiang Province, Assisted Reproduction Unit, Department of Obstetrics and Gynecology, School of Medicine, Zhejiang University, Sir Run Run Shaw Hospital, 3 East Qing Chun Rd, Hangzhou 310020, China; chao_yu@zju.edu.cn; 3 College of Life Sciences, Zhejiang University, 866 Yuhangtang Rd, Hangzhou 310058, China; 4 Department of Dermatology, The Second Affiliated Hospital, Zhejiang University School of Medicine, Zhejiang University, Hangzhou 310029, China

**Keywords:** proteasome, ubiquitination, E3 ubiquitin ligase, deubiquitinating enzyme (DUB), spermatogenesis, meiosis

## Abstract

Spermatogenesis is a prolonged and highly ordered physiological process that produces haploid male germ cells through more than 40 steps and experiences dramatic morphological and cellular transformations. The ubiquitin proteasome system (UPS) plays central roles in the precise control of protein homeostasis to ensure the effectiveness of certain protein groups at a given stage and the inactivation of them after this stage. Many UPS components have been demonstrated to regulate the progression of spermatogenesis at different levels. Especially in recent years, novel testis-specific proteasome isoforms have been identified to be essential and unique for spermatogenesis. In this review, we set out to discuss our current knowledge in functions of diverse USP components in mammalian spermatogenesis through: (1) the composition of proteasome isoforms at each stage of spermatogenesis; (2) the specificity of each proteasome isoform and the associated degradation events; (3) the E3 ubiquitin ligases mediating protein ubiquitination in male germ cells; and (4) the deubiquitinases involved in spermatogenesis and male fertility. Exploring the functions of UPS machineries in spermatogenesis provides a global picture of the proteome dynamics during male germ cell production and shed light on the etiology and pathogenesis of human male infertility.

## 1. Introduction

**Figure 1:**
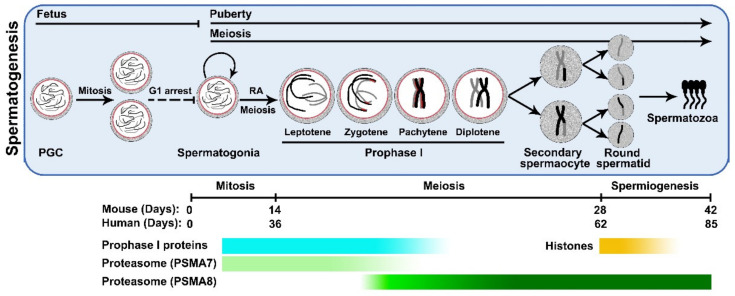
The processes of mammalian spermatogenesis. In males, primordial germ cells (PGCs) are specialized at early stages of fetal development and are arrested at G1 phase. After birth, PGCs differentiate to give rise to spermatogonia in male gonads, or testis. Spermatogonia undergo several rounds of mitosis to produce Asingle (As), Apaired (Apr), Aaligned (Aal), A1–4, intermediate and B types of spermatogonia, progressively. Type B spermatogonia are able to enter meiosis in response to the retinoic acid (RA) signalling. Through homologous recombination and synapsis at Prophase I, homologous chromosome segregation at Metaphase I and sister chromatids separation at Metaphase II, one spermatocyte divides to form 4 haploid round spermatids with ploidy reduction. Following the process of spermiogenesis, round spermatids mature to become spermatozoa with the capacity to fertilize MII eggs. The timelines of spermatogenesis in mouse and human are indicated (not to scale). The degradation timing of regulatory proteins involved in meiotic prophase I (Prophase I proteins) and histones as substrates of UPS, as well as the expression pattern of 20S proteasome subunits PSMA7 and PSMA8 are labeled with Gradient bar.

Spermatogenesis, which occurs within seminiferous tubules of male testes, ensures sustainable production of male gametes, the spermatozoa [1]. Generally, this sophisticated process could be divided into three main phases: (i) the mitotic phase that A single (As) spermatogonia undergo several rounds of mitosis to give rise to undifferentiated spermatogonia (A paired and A aligned), differentiating spermatogonia (A1, A2, A3, A4 and intermediate) and ultimately, type B spermatogonia, which are capable of entering meiosis and forming preleptotene spermatocytes; (ii) the meiotic phase that starts with complicated chromosomal behaviors in meiotic prophase I, such as DNA double strand break (DSB) formation and repair, homologous recombination, and synapsis, followed by two rounds of cell divisions to allow the separation of homologous chromosomes and sister chromatids and to produce haploid spermatids; (iii) the postmeiotic phase, or spermiogenesis, that haploid spermatids mature to motile spermatozoa via 8 steps of round spermatids and 8 steps of elongating spermatids [2,3] (Figure 1). 

In mammals, this continuous spermatogenic program, from As spermatogonia to elongated spermatozoa, involves more than 40 steps and extends for months (42 days in mice and 85 days in humans, respectively) [4,5]. Through spermatogenesis, male germ cells experience dramatic changes in morphology, cellular components, genetics as well as epigenetics and therefore require coordinated, sequential functioning of different protein groups, both functional and structural, to ensure stage-specific molecular interactions and cellular events [6]. 

## 2. The Ubiquitination-Proteasome System

In all cell types, precise control of the effective window of a given protein relies largely on multiple machineries that regulate its abundance via tightly and precisely controlled protein synthesis and degradation. In recent years, genome-wide RNA-sequencing (RNA-seq) of purified male germ cells or isolated single cells has provided fruitful knowledge on the transcriptional regulations during mammalian spermatogenesis [7,8]. However, RNA-seq offers barely no information on protein degradation. 

**Figure 2:**
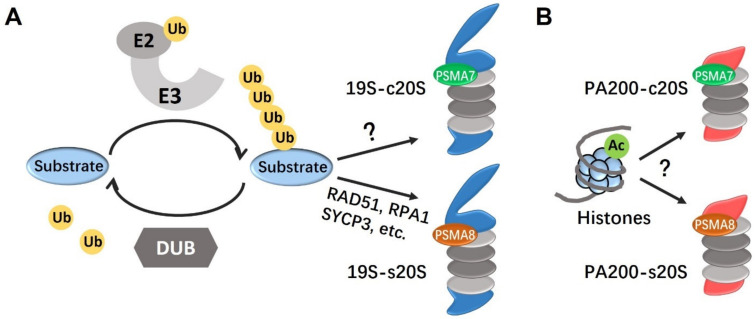
The schematic showing the functions of the UPS to regulate protein homeostasis during spermatogenesis. In male germ cells, the stability of proteins (or substrates here) are tightly regulated by the UPS system, including the E3 ubiquitin ligase that tags substrates for ubiquitination and the DUB that removes ubiquitin (ub) chain from the substrates to recycle both ubiquitin and substrates. Proteasomes are capable of degrading ubiquitin-linked substrates for proteolysis or degradation. So far, there are at least four types of proteasomes in testes (19S-c20S, 19S-s20S, PA200-c20S, PA200-s20S), probably expressed in different stages with distinct functions during spermatogenesis (**A**,**B**). RAD51, RPA1 and SYCP3, which are involved in synapsis and homologous recombination, are largely degraded by 19S-s20S proteasome to mediate meiotic progression; correspondingly, the unique substrates and functions for 19S-c20S proteasome remain to be explored (**A**). PA200 subunit is responsible for the degradation of acetylated histones to promote histone-to-protamine replacement during spermiogenesis; and it remains to be determined whether c20S or s20S is involved in this process (**B**).

The ubiquitination-proteasome system (UPS) plays crucial roles in the regulation of protein degradation, and in some cases, protein activity [9,10] (Figure 2). Ubiquitin is a 76-amino-acid polypeptide with diversities only in 2 amino acids from yeast to human. Action of the UPS is initiated by a three-step enzyme cascade, consisted of ubiquitin-activating enzyme (E1), ubiquitin-conjugating enzyme (E2), and ubiquitin ligase (E3). This cascade transfers ubiquitin to the substrates via formation of covalent bonds between C-terminal glycine residues (Gly76) on the ubiquitin as well as lysine residues on the substrates or ubiquitin. The recognition between E3 ubiquitin ligases and their substrates is critical. In most cases, poly-ubiquitinated substrates are then recognized by the proteasomes for proteolysis and degradation, which terminates the function of the substrates [11]. However, in some cases, the ubiquitin chain could also be removed by deubiquitinating enzymes (DUBs), which recycles both functional proteins and ubiquitin [12]. 

Proteasomes, comprised of a 20S core particle (CP) and one or two regulatory particles (RPs), are central executors to the UPS and protein degradation [13]. The 20S CP is a 28-subunit complex with the molecular weight of around 750 kDa. These subunits are arranged as four stacked rings, two identical outer α rings and two identical inner β rings, forming a barrel-like structure. Each ring, either α or β, contains seven evolutionally-conserved subunits, i.e., α1–α7 and β1–β7, and among which, β1, β2 and β5 subunits possess the caspase-like, trypsin-like and chymotrypsin-like proteolysis activities, respectively [14,15]. This is the most common 20S proteasome species and is termed constitutive 20S proteasome, or c20S. In immunocytes or cells under inflammatory conditions, the three catalytic β subunits are replaced by β1i, β2i and β5i subunits, forming the so-called immune-20S proteasome, or i20S, which facilitates antigen presenting [16]. Recently, several studies uncovered the physiological functions of a male germ cell-specific spermato-20S proteasome, or s20S CP, in the regulation of homologous recombination and meiotic progression [17,18,19]. In the s20S CP, the α4 subunit is replaced by its analogue, α4s [20]. Accumulative evidences have shown that the 20S CP alone has proteolysis activity to degrade unfolded or disordered proteins under stress conditions [21,22]. 

However, the proteolytic activity and specificity of proteasomes are significantly increased by adding RPs to the 20S proteasome [13]. The RPs are attached to the α rings of the 20S CP, acting as the gates of the proteasome to recognize certain proteins for the subsequent proteolysis and meanwhile to prevent the degradation of other proteins. 19S RP is the most common RP and is made up of 17 subunits with a molecular weight of 700 kDa. The 19S RP binds to one or two ends of the 20S CP to form the 26S proteasome, which is the most abundant proteasome isoform responsible for the degradation of majority of poly-ubiquitinated proteins [23]. Besides the 19S RP, additional RPs are found to be associated with the 20S proteasomes, such as the PA28 RPs in immunocytes and the PA200 RP in male germ cells [24,25].

Events of proteasome degradation are common and crucial to spermatogenesis [26]. First of all, male germ cells have diverse proteasome isoforms, which possess specialized activities and exert their specific roles in protein degradation at distinct stages of spermatogenesis. Secondly, massive proteomic alterations take place during spermatogenic processes. For example, H2B dissociation from the chromosomes upon ubiquitination is important for both homologous recombination and meiotic double strand break (DSB) repair [27]. The dissociation of ubiquitinated H2A and H2B also ensures successful histone-to-protamine replacement during spermatid chromatin condensation [28]. In addition to histones, the tremendous loss of mitochondria in elongating spermatids is mostly carried out by the 26S proteasome as the form of enfolded cytoplasmic lobe, which contains both mitochondria and other organelles [29,30]. Single mitochondrial degradation also occurs when its outer membrane proteins (e.g., Mitofusins) are ubiquitinated and presented to the 26S proteasome [31]. Dissociation of mitochondrial outer membrane exposes its inner contents (e.g., prohibitin) for further degradation [30]. Finally, mutations of many UPS-related genes, including genes encoding E3 ubiquitin ligases, DUBs and proteasome components, results in dysregulated spermatogenesis and male infertility in humans and mice. In this review, we summarize the functions of the UPS components in spermatogenesis and intend to draw a comprehensive schematic of protein degradation during this process. 

## 3. Proteasomes in Male Germ Cells

Testis specificity of the proteasomes relies largely on its unique isoforms of RP and CP subunits. In *Drosophila*, nearly one third of the 20S CP subunits possess alternative isoforms, the majority of which are solely expressed in testis [32]. For example, the α3 isoform, Prosα3T; the α4 isoform, Prosα4T1 (Pros28.1A) and Prosα4T2 (Pros28.1B); and the α6 isoform, Prosα6T [32,33,34]. These 20S isoforms are exclusively expressed in *Drosophila* testes from meiotic to post-meiotic germ cells, and gradually replace the constitutive isoforms to exerts their biological functions. Prosα6T, for instance, plays a pivotal role in sperm individualization and nuclear maturation, and therefore, *Drosophila* carrying *Prosα6T* mutation show disrupted individualization complexes and abnormal nuclear bundles [33].

Mammalian testis also has its distinctive features in proteasome composition, mainly due to the expression of male germ cell-associated proteasomal components, α4s and PA200, which are encoded by *Psma8* and *Psme4*, respectively (Figure 2). A very recent preprint has revealed the complexity of proteasome composition in developing male germ cells by mass spectrometry (MS) [35]. Dramatic dynamics have been observed throughout the whole process of spermatogenesis, such as an increase in the amount of 20S proteasomes in spermatids, replacement of α4 (PSMA7) by α4s (PSMA8) in spermatocytes, as well as assembly of PA200-20S proteasomes in spermatocytes and spermatids. Therefore, it is of great significance to understand the stage-specific proteasome composition and relevant degradation events during spermatogenesis.

### 3.1. PSMA8

In mitotic spermatogonia and spermatocytes before the pachytene stage, around 98% 20S CP are made up of PSMA7, the constitutive α4 subunit [17,35]. However, from the pachytene stage onwards, the composition of 20S proteasomes are gradually shifted to PSMA8-associated s20S proteasomes [17,20]. This transition, from c20S to s20S, is mainly due to the expression of PSMA8 in pachynema. However, in PSMA8-null spermatocytes beyond the pachytene stage, the amount of c20S proteasomes is significantly decreased, suggesting that this c20S to s20S transition is an active process, through disassembly of the c20S and subsequent reassembly of the s20S (Figure 1) [17,19,35]. This type of male germ cell-specific s20S CP persists from meiotic spermatocytes to haploid spermatids and is required for proper protein degradation in these cells. Several recent studies have investigated the physiological and biochemical functions of PSMA8, or s20S CP, in spermatogenesis [17,18,19]. Although the exact function of PSMA8 in spermatogenesis remains controversial, one consensus that has been reached is its indispensability for Meiosis I (MI) progression. Spermatocytes of PSMA8-deficient mice show delayed entry into metaphase I and are finally arrested at this stage, resulting in few, if any, spermatids and mature sperm in the seminiferous tubules or epididymis, and ultimately, male infertility [17,18,19]. 

Progression from Prophase I to Anaphase I can be affected by a series of events that take place during Prophase I, such as assembly and disassembly of the synaptonemal complex (SC), generation and repair of meiotic DSBs, homologous recombination, and cell cycle control [3]. Most studies suggest that meiotic DSB generation and repair, synapsis, homologous recombination as well as crossing-over formation are less affected upon PSMA8-deletion [17]. This conclusion was supported by immunofluorescent (IF) staining of many protein markers (RPA proteins, ZMM proteins, MLH1, etc.), which are comparable in WT and PSMA8-deficient spermatocytes at similar developmental stages. 

Efforts have been made to identify the direct targets of s20S CP, through methodologies of Western blotting (WB), IF staining and MS. In consistent with its function in protein proteolysis, the level of protein ubiquitination is accumulated in PSMA8-deleted testes [17,18]. Moreover, PSMA8-deficient male germ cells are incompetent to degrade certain Prophase I proteins (Figure 2). For example, RAD51 and RPA1 are highly expressed from zygotene to early-pachytene stages, but supposed to be degraded at late-pachytene and diplotene stages in normal spermatogenesis. However, these two proteins are found accumulated in PSMA8-deficient spermatocytes at late-pachytene and diplotene stages [17]. According to another study, PSMA8 is localized to the central region of SC, which is in consistent with a previous study demonstrating the chromosomal localization of proteasomes [18,36]. Correspondingly, abnormal accumulation of the transverse filament protein SYCP1 and the axial element protein SYCP3 is found in PSMA8-deficient spermatocytes. In a third paper, the authors proposed the functions of s20S CP, in complex with PA200, in the degradation of core histones upon DNA damage and during spermiogenesis [19]. Additionally, maturation-promoting factor (MPF) complex, consisting of cyclin-dependent kinase 1 (CDK1) and cyclin B, is central to cell cycle regulation. MPF activity is required for metaphase entry, however its inactivation is crucial for metaphase-anaphase transition. PSMA8 deficiency leads to elevated MPF activity, which is consistent with the phenotype of MI arrest [17,18]. 

Further experiments suggest that PA200 is co-immunoprecipitated with PSMA8. PSMA8 deficiency causes significant decrease in PA200 level, as well as the intensity of 20S catalytic subunits β1, β2 and β5, which ultimately results in abortive proteasome assembly [17,18]. Since the majority of 20S CPs in meiotic and post-meiotic male germ cells possess PSMA8 instead of PSMA7 and s20S associates with PA200, it was proposed that PA200-s20S is the main proteasome isoform functioning in protein degradation in spermatocytes and spermatids. However, another study found that other than PSMA8, more PSMA7 protein fragments are recovered in anti-PA200 immunoprecipitants, while on the other hand, s20S CP preferentially binds to the 19S RP, suggesting a controversial composition of proteasome isoforms in male germ cells [35]. This selectivity of combinations between RPs and CPs might be explained by the domain flexibility between PSMA7 and PSMA8. Nevertheless, the underlying mechanisms determining the differences between c20S and s20S CPs and the specificity of s20S CPs to RPs and protein substrates need further investigation.

### 3.2. PA200

PA200 is encoded by *Blm10* in budding yeast, *PSME4* in human and *Psme4* in mouse, and is first identified as a 200-kDa monomeric novel proteasome activator in the nucleus [37]. PA200 associates with 20S CP in both bovine testes and HeLa cells and forms foci following irradiation, suggesting its role in DNA repair and chromatin remodeling [37,38]. Further studies suggest that the PA200 RP exhibits high testis preference and is associated with spermatogenesis and male sub-fertility by enforcing the degradation of core histones via an acetylation-dependent manner, during DSB repair and histone-protamine replacement [25,39]. 

In bovine testes, nearly 90% of the proteasomes contain PA200, while usually lower than 10% proteasomes are capped with PA200 in other tissues, for example, muscle [39]. PA200 deficiency causes significant abnormality in mouse spermatogenesis. PA200-deficient testis exhibits polynucleated giant germ cells, insufficient spermatogenesis, spermatic phagocytosis and accumulation of residual bodies [25,39]. Histone-protamine replacement is a hallmark of spermiogenesis and enables the compaction of sperm DNA into a higher architecture [40]. Despite the constitutive UPS pathway, core histone degradation during spermatogenesis can also be achieved by the testis-specific PA200-proteasome in an acetylation-dependent manner (Figure 2). Unlike the 19S RP, PA200 cannot recognize ubiquitin-linked protein substrates. Instead, PA200 contains an acetyl-lysine binding bromodomain (BRD) capable of recognizing acetylated histones [39]. The C-terminal residues of PA200 anchor to the binding pocket at the α1–α2 interface of 20S CP in the pattern of PA200-20S or PA200-20S-PA200 [39,41,42]. Binding of PA200 to acetylated substrates causes conformational rearrangements of the α rings, creating an open channel for substrate entry [42]. Changes of the α rings further extend to β subunits, resulting in increased trypsin-like, caspase-like and chymotryptic activities [35,42]. Consequently, large amount of H2A, H2B and H4 are found retaining in both round and elongating spermatids, suggesting PA200-deficient germ cells are incapable of histone degradation. The excessive histones can decrease genome stability and DNA damage resistance, so that the PA200-deficient testis reflects strong apoptotic and necrotic signals in both meiotic spermatocytes (mainly at and after the pachytene stage) and post-meiotic spermatids [25].

There are two pivotal stages of spermatogenesis which largely depend on acetylated histone dissociation from the nucleosomes, and are therefore vulnerable to PA200 deficiency. Besides the histone-protamine replacement during spermiogenesis, in meiotic Prophase I DSBs trigger the acetylation of core histones and their subsequent recognition and degradation by PA200-proteasomes [39]. Degradation of core histones from the DSB loci leaves space for the recruitment of DNA damage repair-related proteins. However, this point of view is controversial since some studies have reported that PA200 knockout mouse embryonic stem cells exhibit no significant impairment of their DSB repair ability upon DNA damage [38,41]. Besides, both lymphocyte development and immunoglobulin class switching, which depend on the generation and repair of DSBs, are not disrupted in mice with conventional PA200 knockout [25,37,38]. Moreover, PA200 also incorporates into the constitutive 26S proteasome, generating PA200-20S-19S hybrids [39,43]. This type of proteasome is supposed to be involved in the degradation of both ubiquitin- and acetyl-labelled substrates. 

However, according to another study, at any stage of spermatogenesis (spermatogonia, spermatocytes and spermatids), the 19S RP is the main RP [35]. Even in the most PA200-abundant spermatocytes and spermatids samples, PA200-proteasomes only represents around 5% of the total proteasomes. Moreover, as stated in the PSMA8 section, 19S RP mostly associates with PSMA8 while PA200 has higher or similar affinity to PSMA7 in testis [35]. These biochemical evidences demonstrate that diverged proteasome species (including at least PA200-s20S, PA200-c20S, 19S-s20S) function simultaneously in a single cell at the same or different stages to regulate protein homeostasis. The specificity and activity of these proteasome species will be studied to illustrate the protein degradation events in spermatogenesis. Because most of the current studies were performed with mixed germ cell pools, it might be informative to reinvestigate or further investigate these results with purified synchronized male germ cells from each distinct stage.

## 4. E3 Ubiquitin Ligases in Spermatogenesis

Spermatogenesis requires efficient actions of E3 ubiquitin ligases which are master regulators of UPS to mediate protein turnover. As the last step of the enzymatic cascade, E3 confers substrate specificity and is of high abundance and diversity during spermatogenesis. Till now, at least 73 different E3 have been found highly or uniquely expressed in testis, with some of them having identified substrates and functions in spermatogenesis, such as CUL2, CUL4, UBR2 and RNF8 (Table 1) [44]. However, substrates of most testis-specific E3 ligases are still unclear so that the current understanding of their potential functions remains largely speculative and more comprehensive research are required in the future.

### 4.1. Cullin-Ring Ligase Family

Cullin-RING Ligases (CRLs) family of multi-subunit E3 ligase complexes is the largest E3 ligase family [97]. In CRL complex, Cullin protein is the scaffold protein, recruiting substrate-targeting modules on its N-terminal for substrate-targeting specificity and RING finger proteins, usually ROC1/2, on its C-terminal for E2 combination. The diversity of CRLs depends mainly on the substrate-targeting module, i.e., the adaptor protein and the substrate receptor. The mammalian Cullin protein family comprises CUL1 to 7, as well as PARC. These CRLs are involved in multiple biological processes like cell-cycle control, DNA replication and developmental regulation [98]. Among them, CRL1, CRL2, CRL3, CRL4A and CRL4B have been identified to be spermatogenesis-related. 

The CRL1 complex, or SCFβ-TrCP, is the most known E3 ubiquitin ligase and is expressed ubiquitously in all cell types. This E3 complex comprises the scaffold protein CUL1, the adaptor protein SKP1 (S phase kinase-associated protein), the substrate receptor β-TrCP and the E2-interacting protein ROC1/2. There are two paralogs of β-TrCP in mammals, β-TrCP1 (BTRC) and β-TrCP2 (FBXW11) [69]. β-TrCP1 is expressed in spermatogonia at medium level but highly expressed in spermatocytes. In contrast, β-TrCP2 shows medium expression level in spermatogonia and low expression level in meiotic cells. β-TrCP1 deficiency in mice leads to prolonged and abnormal meiosis as indicated by accumulating MI spermatocytes and multinucleated spermatids [70]. In these β-TrCP1-deleted spermatocytes, stabilization of EMI1 is identified. EMI1 is a *bona fide* substrate of β-TrCP1, regulating metaphase to anaphase progression in both mitosis and meiosis via its inhibitive effects on the anaphase promoting complex/cyclosome, or APC/C [99]. While to progress through and exit from metaphase, APC/C needs to be reactivated, depending on EMI1 degradation. However, in spermatocytes lacking β-TrCP1, APC/C complex is inhibited by the accumulated level of EMI1. On the other hand, β-TrCP2 deficiency alone doesn’t cause any defects in spermatogenesis since β-TrCP1 is preserved at sufficient functional levels in all types of cells for compensation [69]. Double deletion of β-TrCP1 and β-TrCP2 leads to severe testis abnormality as indicated by absence of spermatocytes, spermatids and mature sperm. Surprisingly, these mice show dislocated spermatogonia in their seminiferous tubules, which is not observed in mice carrying either single deletion [69]. Snail1 is another substrate of β-TrCP, transcriptionally controlling the level of E-cadherin [100]. Deficiency of β-TrCP1 and β-TrCP2 results in a significantly increased level of Snail1 in these dislocated spermatogonia. As a consequence, accumulated Snail1 reduces E-cadherin and thereby disrupts the integrity of adherent junctions and the trafficking of differentiating cells from the basement of seminiferous tubules to the lumen [69]. 

Similar to the SCF complex, CRL4 complex is made up of the scaffold protein CUL4A/B, the adaptor protein DDB1 (DNA damage binding protein 1), the substrate receptor (DDB1- and CUL4-associated factors, DCAFs) and ROC1/2 [101]. CUL4A is predominantly expressed in primary spermatocytes at the pachytene and diplotene stages [45]. γH2AX indicates the sites of DSBs in meiotic prophase I and can be only detected on XY chromosome pairs in pachytene and diplotene spermatocytes. However, CUL4A deficiency leads to persistent γH2AX signals in mouse pachytene spermatocytes [46]. Similarly, foci of the late homologous recombination protein, MLH1, which are supposed to disappear from diplotene, remain detectable on chromosome pairs of diplotene spermatocytes [45]. As a consequence, significant decrease of post-meiotic spermatids and accumulation of malformed meiotic spermatocytes are evident in *Cul4a* knockout testes [45,46]. In contrast to CUL4A, CUL4B is expressed in spermatogonia and spermatids, predominantly regulating post-meiotic differentiation to guarantee sperm motility [45]. Although CUL4B-deficient mice is still proficient in producing spermatids, most of their spermatozoa possess low motility or become completely immotile, indicating normal meiosis but impaired post-meiotic specification [48]. Diminished mitochondrial activity with low level of ATP production and missing, superabundant or misshapen microtubule doublets on the flagella axonemes are found in spermatozoa derived from CUL4B-deficient mice. DDB1 is the adaptor protein and deletion of which abolishes the activity of all CRL4 complexes [101,102]. Strikingly, conditional deletion of DDB1 in male germ cells with a *Ddx4-Cre* results in complete loss of male germ cells [103]. DCAF12 is one of the substrate receptors of the CRL4 complexes and directly interacts with the Moloney Leukemia Virus 10 (MOV10) [47]. DCAF12 deficiency leads to decreased sperm counts accompanied by elevated level of MOV10. MOV10 is a RNA helicase, included in RNA-induced Silencing Complex (RISC), and plays a role in RNA interference and silencing of transposons, virus, and recently duplicated genes [104]. Increased amount of MOV10 results in the abnormal lower expression of two key meiotic proteins, SYCP3 and γH2AX, suggesting that CRL4A-DCAF12 mediates the degradation of MOV10 during spermatogenesis in a UPS dependent manner to maintain proper levels of meiotic-associated proteins. However, the entire substrates of CRL4 complexes in spermatogenesis remain largely elusive.

Besides SCF and CRL4 complexes, CRL2 and CRL3 also exhibit certain roles in spermatogenesis in *C. elegans* or *Drosophila*. In *C. elegans*, CUL2 is highly expressed in germline and participates in multiple developmental processes in complex with distinct substrate receptors [52]. The Leucine Rich Repeat protein LRR-1, a substrate receptor protein of CRL2, is expressed throughout the germline [53]. In testis, CUL2 associates with LRR-1 to assembly the CRL2 complex and acts with the 26S proteasome to degrade HTP-3, which is a meiotic-related protein recruiter in the mitotic zone of the germline, preparing chromosomes for meiosis [52,105]. Normally, HTP-3 expression shows a low-to-high gradient from the most distal germ cells to the most proximal ones. However, dysfunction of the CRL2 complex leads to uniform distribution of HTP-3 along *C. elegans* gonads, resulting in premature meiotic spermatocytes [105].

*Drosophila* has four CUL3 isoforms and one of them is expressed exclusively in male germ cells (CUL3testis), playing an essential role during post-meiotic differentiation. CUL3testis combines with its adaptor protein Klhl10 and targets dBruce, a caspase inhibitor for degradation [49,50]. This CRL3 E3 ubiquitin ligase complex thereby ensures active function of apoptotic proteins to eliminate superfluous cytoplasm as well as unwanted organelles during post-meiotic differentiation. In mice, CUL3 emerges from post-meiotic differentiation step 9 and keeps detectable through the remaining stages of this process [50]. However, the specific roles of mammalian CUL3 during spermatogenesis remain unsolved since CUL3 deficiency in mice unexpectedly leads to early embryonic lethality by interfering cell cycle progression [51]. 

### 4.2. Other E3 Ubiquitin Ligases

Besides the CRLs family, many other E3 ubiquitin ligases also participate in the regulation of spermatogenesis. In addition to be regulated by the SCF-EMI1 ubiquitination pathway, APC/C itself is an E3 ubiquitin ligase complex and mediates poly-ubiquitination of substrates for proteasomal degradation [106]. During mitosis, APC/C associates with the spindle apparatus to promote proper separation of sister chromatids by mediating cyclin B1 and securin degradation [107]. In addition, APC/C functions to degrade proteins that are necessary for S phase and inactivation of APC/C has been proposed as the commitment point for cell-cycle entry [108]. It is likely that these functions also apply to meiosis, albeit not yet fully characterized. However, a unique role of APC/C in meiosis has been proposed as mediating piRNA-triggered PIWI destruction in late spermatids (both elongating and elongated spermatids) [60]. PIWI-interacting RNA (piRNA) is a novel germline-specific small noncoding RNA and cooperates with PIWI to maintain genome integrity by silencing transposons [109]. Spermatocytes and round spermatids show high level of piRNA/PIWI, while this level begins to decrease in late spermatids, and is ultimately eliminated in mature sperm. Knockdown of endogenous APC/C abolishes PIWI ubiquitination and degradation in late spermatids, resulting in maturation stagnation of these cells [60]. Besides, piRNA is synchronously degraded with PIWI in late spermatids, suggesting a feedforward mechanism for their elimination. That is, piRNA binds to PIWI during late spermatogenesis, causing a conformational change of PIWI, which facilitates its binding to APC/C. Meanwhile, PIWI degradation in turn leaves the piRNA unprotected and therefore primed for elimination [60].

SIAH1A confers cell growth arrest in somatic cells, while in male germ cells, it plays an important role to promote spermatogenesis progression beyond MI [110]. Siah1a deficiency in mice induces severe impact on spermatogenesis characterized by large amounts of spermatocytes accumulated at metaphase to telophase of meiosis I. Besides, anaphase cells are largely bi- or multi-nucleated because of incomplete chromosome segregation [66]. Since ubiquitin-dependent degradation of anaphase inhibitors such as securin is crucial for the transition from metaphase to anaphase [111], it is likely that E3 ubiquitin ligase SIAH1A also targets these proteins as substrates to facilitate anaphase entry. 

HEI10 is an E3 ubiquitin ligase important for the regulation of homologous recombination during pachytene [68]. Following DSB repair mediated by RAD51, HEI10 mediates the degradation of CCNB3 to free CDK2. In HEI10 mutant mouse spermatocytes, homologous recombination initiates normally with proper DSB formation and repair. However, these spermatocytes fail to form chiasmata which leads to premature homolog separation, and ultimately undergo meiotic arrest at MI or apoptosis [112]. 

RNF8, or RING finger protein 8, is essentially a DNA damage response (DDR) protein that ubiquitinates histone H2A and H2AX to facilitate downstream recruitment of DDR factors to DSB-flanking chromosomes [55]. In pachytene spermatocytes, ubiquitinated H2AX (ubH2AX) is significantly enriched in the XY body, a process known as meiotic sex chromosome inactivation, or MSCI [113]. RNF8 deficiency leads to severe loss of ubH2AX in the XY body, indicating a role of RNF8 in ubiquitinating H2AX during this process. However, neither XY body formation nor the subsequent meiosis is affected upon RNF8 deficiency, indicating that RNF8 may facilitate but is not necessary for MSCI or meiosis. In contrast, RNF8 is indispensable during post-meiotic differentiation, when nucleosome removal occurs accompanied by H2A and H2B ubiquitination [55]. RNF8 deficiency in mice leads to reduced ubH2A and ubH2B in elongating spermatids as well as impaired histone-protamine transition. Sperm production in RNF8-deficient mice is significantly impaired with accumulation of defective sperm possessing malformed sperm heads [55]. Notably, although RNF8 itself has no acetyltransferase activity, it is supposed that RNF8 indirectly regulates mammalian acetyltransferase MOF through ubH2A and ubH2B. Therefore, RNF8 deficiency leads to decreased MOF level as well as H4 acetylation [114]. RNF8 also participates in epigenetic modification in post-meiotic spermatids. One study has shown that through H2A ubiquitination, RNF8 is able to achieve active epigenetic modifications such as H3K4 dimethylation (H3K4me2), which further activate escaped genes from silenced sex chromosomes in post-meiotic spermatids [58].

UBR2 also targets histone H2A for ubiquitination conjugation. UBR2 primarily localizes to the unsynapsed chromatin regions where it transfers ubiquitin from its E2 enzyme HR6B to histone H2A, mediating meiotic silencing of unsynapsed chromatin (MSUC) [64]. Furthermore, UBR2-mediated histone ubiquitination is linked to a pachytene checkpoint. UBR2 deficiency impairs multiple meiotic processes such as homologous recombination, MSUC and synapsis, which trigger meiotic arrest through the pachytene checkpoint system [61,62,65]. 

## 5. Protein Deubiquitination in Spermatogenesis

Deubiquitinases (DUBs) are cysteine proteases, which remove and recycle ubiquitin. Till now, more than 100 DUB enzymes have been identified in human [115]. DUBs participate in various biological processes such as apoptosis, cell cycle progression, and protection of proteins from degradation. DUBs are classified into six catalogues: USP (ubiquitin-specific processing proteases), UCH (ubiquitin C-terminal hydrolases), JAMM (Jab1/Pab1/MPN domain-containing metalloenzymes), OUT (Otu-domain ubiquitin aldehyde-binding proteins), MCPIPs (Monocyte chemotactic protein-induced proteases) and Ataxin-3/Josephin [116]. Some DUBs have been demonstrated to play a requisite role during spermatogenesis, which largely belong to the USP and UCH family (Table 2) [115]. 

USP7 localizes preferentially to the XY body in early pachytene spermatocytes but gradually decreases its level as meiosis progresses, which is highly concurrent with the expression manner of SCML2, a testis-specific polycomb protein [119]. Biochemically, USP7 forms a complex with SCML2 to counteract histone H2A ubiquitination in the XY chromatin during meiosis. In SCML2-deficient mice, USP7 is absent from H2A ubiquitination sites, leading to augmented H2A monoubiquitination, which in turn causes spermatogenic impairments characterized by massive pachytene spermatocyte apoptosis [119]. Though the direct effects of USP7 have not been studied yet, it can be drawn from this study that localization of USP7 to the XY body relies on SCML2 and these two proteins cooperate to regulate male meiosis, most likely trough mediating transcriptional silencing. 

USP8 is expressed in both brain and testis and interacts with MSJ1, STAM2, EEA1 and VSP54 [121]. In mouse testis, USP8 shows remarkable increase during post-meiotic differentiation and forms small plaques. USP8 localizes specifically to the nuclear envelope of round spermatids, as well as the centrosome and acrosome vesicle of elongating spermatids [120]. In mouse spermatids, USP8 associates with MSJ1 and the 20S CP to move towards the developing acrosome and centrosome [121,145]. STAM2 gives rise to the endosomal-sorting complex ESCRT-0 and EEA1 is an early endosome antigen. Colocalization of USP8 with STAM2 and EEA1 is found at acrosomal vacuole and acrosome surface, respectively [146]. VSP54 is responsible for retrograde transport from early endosomes, and during post-meiotic differentiation, it follows the same migration trajectory as USP8 until complete acrosome formation is achieved [147]. Taken together, these results suggest that USP8 is a key participant of the endosome pathway during acrosome generation.

USP2 is restrictedly expressed in elongating spermatids, possessing speculative function during post-meiotic differentiation. USP2 deficiency in mice leads to defective sperm whose motility is highly vulnerable to environmental changes [118]. Besides, these sperm exhibit poor fertilization capacity due to failure in binding or penetrating to the zona pellucida. USP14 is important for post-meiotic differentiation. In *Drosophila*, USP14 deficiency impairs spermatid individualization, during which syncytial spermatids are separated into individual cells [128]. USP14 deficiency causes loss of synchronization and abnormal distribution of the actin cones. Besides, USP14-deficient mice exhibit significantly reduced sperm counts as well as severe sperm malformation such as multiple nucleus, missing of sperm head or dual sperm tails [127]. 

Mammalian USP9X is the functional orthologue of drosophila deubiquitinating enzyme fat facets (Faf), which interacts with and impedes the degradation of a DEAD-box RNA helicase Vasa, a highly-conserved marker for germ cells [148]. USP9X is predominantly expressed in spermatogonia and weakly expressed in early spermatocytes before pachytene stage. USP9X-deficiency in mouse germ cells leads to male infertility with various abnormalities along the progression of spermatogenesis: reduced number of spermatocytes, degenerated spermatids with residual body-like structures, as well as aberrantly retained mature spermatozoa in seminiferous tubules [126]. However, the maintenance and proliferation of spermatogonia were less affected in USP9X-deficient testes, indicating a critical role of USP9X since mitosis-to-meiosis transition in male germ cells.

*USP26* has been considered as a potential infertility gene due to its restricted expression in mammalian testis and X-chromosome-localization (single copy in males) [149,150,151]. Several studies have reported the polymorphisms in *USP26* associated with non-obstructive azoospermia or asthenozoospermia, suggesting a causal relationship with human male infertility. However, most of the identified *USP26* polymorphisms can’t disrupt its enzymatic function [152]. In addition, *USP26* is indispensable for mouse fertility in both sexes [153]. It has recently been found the effects of *USP26* mutation on male fertility dependent on the genetic background of mice [154]. *USP26* mutants in DBA/2, rather than C57BL/6 background exhibit impaired spermatogenesis with obvious deficiency and malformation of spermatozoa, resulting in infertile or sub-fertile males. These results implicate that strain-specific genetic components interact with *USP26* mutations to interfere spermatogenesis; such components may also exist in human but further investigations are needed.

UCHL3 expression exhibits differentiation-dependent pattern during spermatogenesis with minimal amount in spermatogonia but to a sequentially increasing extent in meiotic pachytene spermatocytes and post-meiotic spermatids [141]. Besides, a later study has detected intensive UCHL3 expression in sperm acrosomes and flagella, suggesting the role of UCHL3 in regulating both meiosis and post-meiotic differentiation [155]. This study also examines the UCHL3 level in patients with asthenozoospermia (A) and oligoasthenozoospermia (OA) who possess malformed spermatozoa as characterized by small or absent acrosome, as well as atrophic or distorted tail. According to the results, OA patients show significantly lower UCHL3 content and activity as compared to normozoospermia controls; UCHL3 condition is slightly better in A patients but still far from normal level [155]. Meanwhile, it is proposed that the amount and activity of UCHL3 are positively related to a series of fertility indicators including sperm counts, sperm concentration and sperm motility, highlighting the importance of this DUB during spermatogenesis. 

UCHL1 shares high sequence similarity with UCHL3, however, its distribution pattern in male germ cells is totally different, indicating distinct function during spermatogenesis [156]. Predominant UCHL1 expression is found in spermatogonia as well as in Sertoli cells. The level of UCHL1 precisely determines spermatogonia fate of either self-renewal or meiotic differentiation as indicated by two markers Plzf and c-Kit, respectively [156]. In mouse testis, UCHL1 overexpression leads to drastic loss of post-meiotic germ cells and a large amount of arrested pachytene spermatocytes retards in seminiferous tubules which further undergo apoptosis [138]. On the contrary, UCHL1 deficiency significantly increases the number of spermatogonia and preleptotene spermatocytes while immensely inhibits germ cell apoptosis during the first round of spermatogenesis. Additionally, these mice have reduced sperm motility and more abnormal spermatozoa even though complete sterility is not observed [139]. Taken together, UCHL1 and UCHL3 express strongly but reciprocally during spermatogenesis, where the former mediates fate determination of spermatogonia as well as elimination of defective spermatozoa to maintain testicular homeostasis, while the latter facilitates post meiotic maturation to produce fertilization-competent spermatozoa.

Besides the DUBs in USP and UCH families, CYLD (cylindromatosis) is also found to be required for spermatogenesis by regulating early wave of germ cell apoptosis mainly in spermatogonia, a requisite process to eliminate excessive germ cells and thereby maintain the balance between germ cells and Sertoli cells [157]. CYLD directly deubiquitinates the receptor-interacting protein 1 (RIP1), to prevent the activation of IKK and NF-𝜅B signaling and the expression of downstream anti-apoptotic genes, ultimately promote cell apoptosis [144]. Loss of CYLD leads to reduced spermatozoa, failure in radial organization of round spermatids, as well as malformation of acrosomes in elongating spermatids. On the contrary, spermatogonia and early spermatocytes are aberrantly accumulated in CYLD-deficient seminiferous tubules.

## 6. Conclusions and Perspective

For a prolonged and highly ordered physiological process such as spermatogenesis, the precise control of protein homeostasis to ensure the functioning of certain protein groups at a given stage and the inactivation of them after this stage (actively or passively) is critical for the step-by-step progression. In this review, we summarized the biochemical and physiological functions of the UPS components in male spermatogenesis, including the diverse proteasomes that target ubiquitinated proteins for proteolysis, the E3 ubiquitin ligases that tag their substrates for ubiquitination, as well as the DUBs that remove ubiquitin chains from the substrates. We propose further investigation on the underlying mechanisms of UPS-mediated protein homeostasis during spermatogenesis, in order to reveal the unique protein degradation events in male germ cells, interpret the specificity of testis-associated proteasome isoforms in targeting different groups of substrates, and clarify the pathogenesis of human male infertility related to UPS dysfunction. However, most related studies do not take the big picture of the UPS into consideration. For example, some studies only investigate an E3 and sometimes including its substrates but do not analyze the following degradation events, or some other studies focus on a proteasome isoform but omit its substrate specificity and the biochemical uniqueness. The main reason should be the complexity of both the UPS and the spermatogenic procedure, and one way to improve it might be focusing on a certain protein in spermatogenesis, to systematically investigate its expression pattern transcriptionally and translationally, its E3 and DUB, the ubiquitination cascade and the proteasome isoform that proteolyzes it. The other way is to investigate the proteomic alterations during each step of spermatogenesis with advanced techniques, such as quantitative MS and ubiquitination-related MS. 

Since the regulation of protein ubiquitination and degradation is always specific and precise to a narrow time window, the significance of a single degradation event might be underestimated, if the result is drawn with male germ cells of mixed stages. Due to the development of a WIN18,446/RA-based protocol to synchronize spermatogenesis into a “tsunami” [158], it is possible to study proteomic changes with synchronized, pure germ cell samples. And by investigating the specialized physiological process of spermatogenesis, we may reveal the unique regulatory mechanisms of UPS in physiological conditions and broaden our understanding of both UPS and spermatogenesis. 

Around 8–12% of couples worldwide suffer from infertility and male factors contribute to up to 20–30% of the cases independently [159]. Causes of male infertility, such as azoospermia, oligozoospermia, teratozoospermia, etc., have been comprehensively studied in both medical and basic research, which largely influence the therapeutic methods and success rates in IVF-ET (In vitro fertilization and embryo transfer). Regulatory functions of UPS components found in mouse spermatogenesis have been confirmed in humans. For example, PA200-proteasomes are also responsible for histone-protamine transition in human spermiogenesis; deletions of USP9Y have been found in infertile male patients; declined level and activity of UCHL3 cause defects in human sperm count and motility [124,155,160]. Therefore, identification of novel genes in UPS correlating with spermatogenetic failure could unravel the genetic cause of male infertility and promote the prevalence of PGD (Preimplantation genetic diagnosis) before ICSI (Intracytoplasmic sperm injection), ultimately improve the birth quality of human beings.

## Figures and Tables

**Table 1:** Overview of E3 ubiquitin ligases associated with spermatogenesis.

Names	Expression Pattern (In Testis)	Substrates	Mutant Phenotypes	Species	References
CUL4A	Sg, Sc, St (low)	Histone H3, H4; MOV10	Infertility; apoptosis of Sc; decreased St and Sz; abnormal Sz with low motility and defective acrosome formation	*M. musculus*	[45,46,47]
CUL4B	Sg, St	Unknown	Sz with low or no motility	*M. musculus*	[48]
CUL3	St	dBruce	Reduced or eliminated effector caspase activation in St	*D. melanogaster*	[49]
CUL3	St	Unknown	Early embryonic death	*M. musculus*	[50,51]
CUL2	Male germ cells (high in Sc)	HTP-3	Infertility; cell cycle arrest; premature meiotic entry	*C. elegans*	[52,53]
RNF8	Undefined	Histones H2A, H2AX, H2B	Infertility; defective nucleosome removal in St	*M. musculus*	[54,55,56,57,58]
APC/C	Sc, St	PIWI/MIWI	Defective maturation of St	*M. musculus*	[59,60]
UBR2	Testis	Histones H2A, H2B	Infertility; meiotic arrest; defective homologous recombinational repair, MSUC, and synapsis	*M. musculus*	[61,62,63,64,65]
SIAH1A	Testis	Unknown	Infertility; metaphase to telophase arrest during meiosis I; bi- or multi-nucleated anaphase cells	*M. musculus*	[66,67]
Mei4/HEI10	Undefined	CCNB3 (?)	Infertility; failure of crossover formation; meiotic arrest; apoptosis of Sc	*M. musculus*	[68]
SCFβ-TrCP	Sg, Sc	Snail1, Emi1 (?)	β-TrCP1 deficiency: accumulation of metaphase I Sc and multinucleated St; β-TrCP1/2 double deficiency: absence of Sc, St and Sz	*M. musculus*	[69,70]
RAD18/18sc	Testis (high in Sc)	Unknown	Subfertility; defective meiotic DSB repair and MSCI	*M. musculus*	[71,72]
RFP	Male germ cells	Unknown	Undefined	*M. musculus*	[73,74]
Parkin	Undefined	Unknown	Infertility; defective mitochondrial organization and St individualization	*D. melanogaster*	[75,76]
E6-AP	Undefined	Unknown	Subfertility; reduced Sz	*M. musculus*	[77]
ZNF645	Sc, St, Leydig cells	Unknown	Undefined	*H. sapiens*	[78]
ITCH	Testis	Occludin	Subfertility; age-dependent impairment; delayed St development and organization	*M. musculus*	[79,80]
HUWE1/LASU1	Sg, Sc	Histones (?)	Undefined	*R. norvegicus*	[81,82]
HERC4	Sg, Sc, St	Unknown	Subfertility; reduced and abnormal Sz	*M. musculus*	[83]
UBR5/HYD/EDD	Sc, St (low)	Unknown	Rat: Undefined *Drosophila*: Infertility; defects in St elongation	*R. norvegicus* *D. melanogaster*	[84,85,86,87]
TMF/ARA160	Sc, St	Unknown	Infertility; reduced motility and malformation of Sz	*M. musculus*	[88]
RNF4	Undefined	MDC1, BRCA1	Infertility; age dependent testicular atrophy; germ cell depletion	*M. musculus*	[89]
RNF19A	Pachytene Sc, St, Sz	Unknown	Undefined	*R. norvegicus*	[90]
RNF133	St	Unknown	Undefined	*M. musculus*	[91]
RNF149	Male germ cells	Unknown	Undefined	*R. norvegicus*	[84]
RNF151	St	Dysbindin (?)	Undefined	*M. musculus*	[92]
RNF168	Undefined	Unknown	Subfertility or infertility; age-dependent impairment; testis atrophy	*M. musculus*	[93]
MARCH7	Testis (high in St)	Unknown	Undefined	*R. norvegicus*	[94]
MARCH10	St	Unknown	Undefined	*R. norvegicus*	[95]
MARCH11	St	Unknown	Undefined	*R. norvegicus*	[96]

Sg: spermatogonia; Sc: spermatocyte; St: Spermatids; Sz: spermatozoa. “?” indicates further studies are required to verify the substrates.

**Table 2:** Overview of DUBs associated with spermatogenesis.

Names	Expression Pattern (In Testis)	Substrates	Mutant Phenotypes	Species	References
USP2	Ubiquitous (high in elongating St)	Unknown	Subfertility; abnormal aggregation of St	*M. musculus*	[117,118]
USP7	Sc (Early-pachytene)	RNF2	Undefined	*M. musculus*	[119]
USP8	Testis (high in St, Sz)	MSJ1 (?)	Undefined	*M. musculus*	[120,121]
USP9Y	Undefined	Unknown	SNP associated with azoospermia/oligospermia	*H. sapiens*	[122,123,124,125]
USP9X	Sg (high), Sc (low)	Unknown	Infertility; reduced reduced Sc; malformation of St and Sz	*M. musculus*	[126]
USP14	St and Sz	Unknown	Defective St individualization; reduced and abnormal Sz	*D. melanogaster*, *M. musculus*	[127,128]
USP26	Testis	Unknown	Polymorphisms associated with azoospermia/oligozoospermia/asthenozoospermia	*H. sapiens*	[129,130,131,132,133,134]
UPS42	St	Unknown	Undefined	*M. musculus*	[135]
UCHL1	Sg, Sertoli cells	Unknown	Increased Sg; reduced motility and malformation of Sz; resistant to early wave of germ cell apoptosis	*M. musculus*	[136,137,138,139]
UCHL3	Pachytene Sc, St	Unknown	Increased germ cell loss after cryptorchid injury	*M. musculus*	[140,141,142]
UCHL4	Sg	Unknown	Undefined	*M. musculus*	[141,143]
UCHL5	Sc, St	Unknown	Undefined	*M. musculus*	[141]
CYLD	Ubiquitous	RIP1	Infertility; deficiency and malformation of St; attenuates the early wave of germ cell apoptosis	*M. musculus*	[144]

Sg: spermatogonia; Sc: spermatocyte; St: Spermatids; Sz: spermatozoa. “?” indicates further studies are required to verify the substrates.
